# The Control of Developmental Phase Transitions by microRNAs and Their Targets in Seed Plants

**DOI:** 10.3390/ijms21061971

**Published:** 2020-03-13

**Authors:** Jingyi Ma, Pan Zhao, Shibiao Liu, Qi Yang, Huihong Guo

**Affiliations:** 1 College of Biological Sciences and Biotechnology, National Engineering Laboratory for Tree Breeding, Beijing Forestry University, No. 35, Tsing Hua East Road, Haidian District, Beijing 100083, China; y602931596@163.com (J.M.); zhaopan11250528@163.com (P.Z.); yangqi0808@bjfu.edu.cn (Q.Y.); 2 College of Biology and Environmental Sciences, Jishou University, Jishou 416000, China; liushibiao_1@163.com

**Keywords:** plant development, phase transitions, miRNAs, target genes, regulatory role

## Abstract

Seed plants usually undergo various developmental phase transitions throughout their lifespan, mainly including juvenile-to-adult and vegetative-to-reproductive transitions, as well as developmental transitions within organ/tissue formation. MicroRNAs (miRNAs), as a class of small endogenous non-coding RNAs, are involved in the developmental phase transitions in plants by negatively regulating the expression of their target genes at the post-transcriptional level. In recent years, cumulative evidence has revealed that five miRNAs, miR156, miR159, miR166, miR172, and miR396, are key regulators of developmental phase transitions in plants. In this review, the advanced progress of the five miRNAs and their targets in regulating plant developmental transitions, especially in storage organ formation, are summarized and discussed, combining our own findings with the literature. In general, the functions of the five miRNAs and their targets are relatively conserved, but their functional divergences also emerge to some extent. In addition, potential research directions of miRNAs in regulating plant developmental phase transitions are prospected.

## 1. Introduction

The life cycle of seed plants is composed of a succession of distinct developmental phases, mainly including vegetative growth, reproductive growth, seed/embryo set [1]. The vegetative growth can be further divided into juvenile and adult phases [2], and the reproductive growth entails the vegetative-to-reproductive transition and the development of floral organs [3]. The seed/embryo set, as a unique development process of seed plants, also includes multiple developmental phases [4]. In addition, storage organ formation is also a special development process, which involves various developmental transitions such as underground stolon-to-tuber and aerial stem-to-rhizome transitions [5,6,7]. The transitions between developmental phases involve changes in morphological and physiological traits, which may be reflected in organ/tissue size and shape, meristem identity or activity, premature or late-maturity, early or late flowering, etc. [2,8,9,10]. For instance, the juvenile-to-adult transition is featured by the formation of leaf abaxial trichomes and increased leaf length/width ratio and serration in *Arabidopsis* [11]; the vegetative-to-reproductive transition is characterized by the conversion of vegetative shoot apical meristem (SAM)-to-inflorescence meristem, and then inflorescence meristem-to-floral meristem, thereby determining flowering [1].

Cumulative data show that developmental phase transitions can be genetically regulated by microRNAs (miRNAs), which participate in complex genetic networks controlling the development of plants [12,13]. MiRNAs are an extensive class of small endogenous non-coding RNAs of around 21–22 nucleotides in length [13]. The first miRNA to be identified was *lin-4* RNA, which is a key regulator controlling the developmental timing in *Caenorhabditis elegans* [14]. Since the discovery of plant miRNAs in *Arabidopsis* [15], a large number of miRNAs have been continuously identified in plants in recent years [16]. Plant genomes typically possess several hundreds of miRNA genes, many of which exist as families [17,18,19]. MiRNA genes are first transcribed by DNA-dependent RNA polymerase II into primary miRNAs and then cleaved by Dicer-like proteins to generate precursor miRNAs, which are further processed by the Dicer enzyme to form mature miRNAs with potential regulatory functions on the expression of genes [19,20,21]. Current research has revealed that miRNAs usually negatively regulate their target genes by post-transcriptional mRNA cleavage or translational repression [3,22,23,24,25].

**Figure 1:**
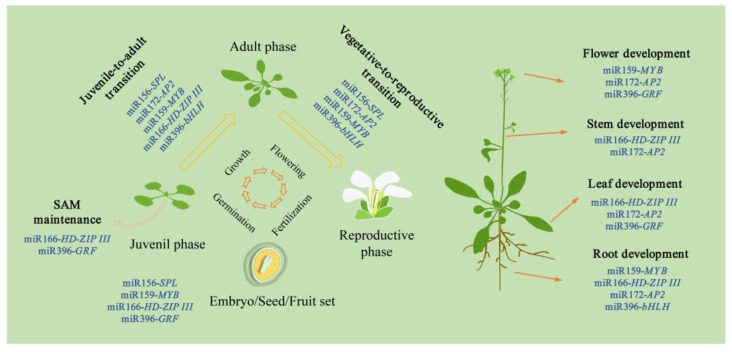
MiRNAs and their targets regulate the developmental phase transitions in plants. MiRNA-target modules not only regulate the juvenile-to-adult and vegetative-to-reproductive transitions, but also control the normal transitions within the formation of tissues/organs, including SAM, root, stem, leaf, flower and embryo/seed/fruit. The functions of the miRNAs, included in Figure 1, have been demonstrated in many plant species.

**Figure 2:**
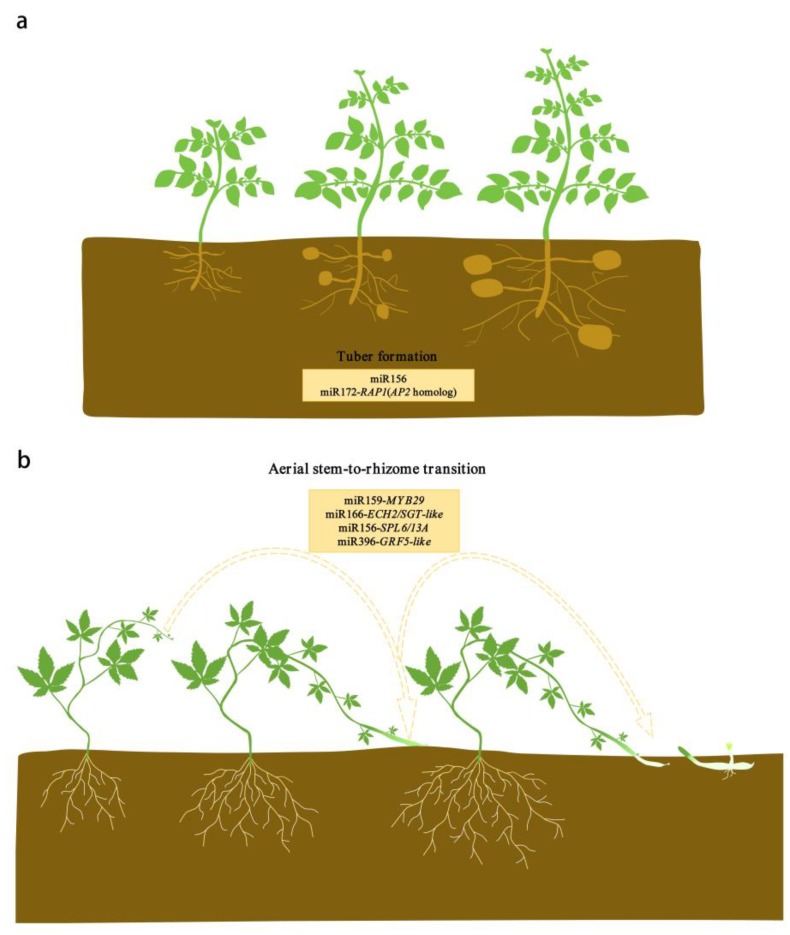
MiRNAs and their targets regulate the formation of storage organs. (**a**) MiR156 and miR172-*RAP1*(*AP2* homolog) module regulate the stolon-to-tuber transition in potato. (**b**) MiR159-*MYB29*, miR166-*ECH2*/*SGT*-like, miR156-*SPL6*/*13A*, and miR396-*GRF5*-like modules are involved in the aerial stem-to-rhizome transition of *Gynostemma pentaphyllum*. In *G. pentaphyllum*, the subapical regions of some aerial stems swell and then drill into the soil to form rhizomes that produce new plants in the next year, which is an adaptive regenerative strategy that enables it to survive during winter.

**Figure 3:**
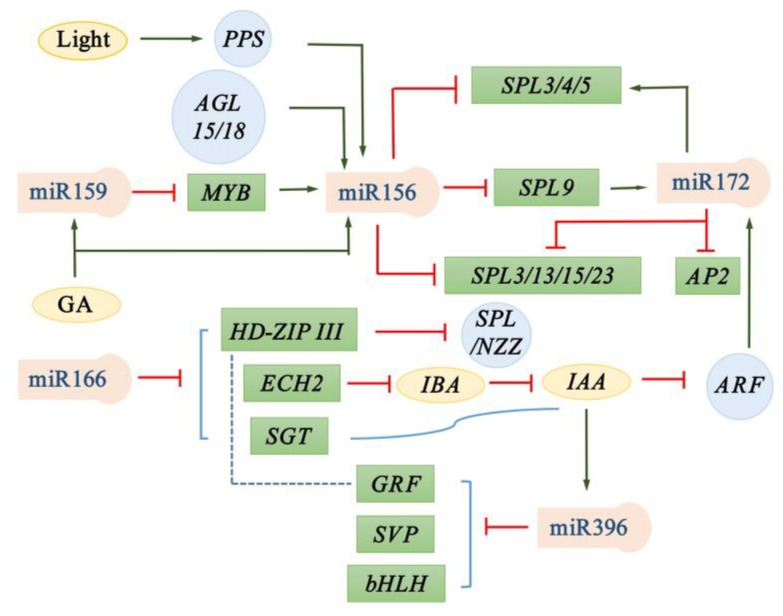
Genetic networks of developmental phase transitions regulated by miRNA-target modules together with their upstream or downstream transcription factors, hormone and light. Arrow represents activation, line with a bar represents repression and dotted line represents possible interactions. MiRNAs are shown in pink boxes and their targets are shown in green ones. Hormones and light are shown in yellow oval frames, and transcription factors are shown in blue round frames.

In recent years, significant progress has been made in exploring the molecular regulation of miRNAs in the developmental phase transitions of plants. It is worth noting that miRNAs were first discovered to be involved in the aerial stem-to-rhizome transition of *Gynostemma pentaphyllum* in one of our recent studies. In this review, we focus on the relatively well-studied miR156, miR159, miR166, miR172, and miR396, and discuss the roles of these five miRNAs and their targets in plant developmental phase transitions, combining our own findings with literature (Figure 1, Figure 2 and Figure 3). Simultaneously, we emphasize the effects of these five miRNAs and their targets on developmental transitions in the formation of storage organs (Figure 2). We also discuss the functional conservation and divergence of these miRNA-target modules in regulating the developmental phase transitions of plants.

## 2. miR159

The MiR159 family, involved in stress tolerance, has been widely known, but it has also been found to play an important role in the developmental phase transitions of plants [26,27,28]. The members of the miR159 family regulate developmental phase transitions by targeting *MYB* transcription factor genes in plants [15,29]. The MiR159-*MYB33* module was originally found to control the transition from vegetative to reproductive in *Arabidopsis*, and elevated miR159 expression of the down-regulated gibberellin (GA)-specific *MYB33* level, which reduced a flowering promoter *LEAFY* activity and thus delayed flowering time [30]. A similar finding was detected in transgenic gloxinia plants overexpressing miR159a, where increased expression of miR159a down-regulated a GA-specific *MYB* gene and exhibited significantly late flowering [31]. In monocot rice, the overexpression of miR159 also silenced an *MYB* gene and resulted in delayed flowering [32]. These findings suggest that miR159 seems to act as a negative regulator in vegetative-to-reproductive phase transition. However, there are several conflicting reports on the involvement of miR159 in the regulation of GA-mediated flowering. In *Arabidopsis*, the overexpression of miR159a had no effect on flowering time but caused anther defects, and the expression of *MYB33* in flowers did not change [33]. Surprisingly, the *mir159ab* double-mutant also delayed flowering, like transgenic plants overexpressing miR159a, but the mutations of their two targets (*MYB33/MYB65*) did not affect flowering time in *Arabidopsis* [34]. In a recent report, the decreased miR159 level regulated by miR167 up-regulated the expression of an *MYB33*-like gene, which was in line with the late flowering of tobacco [35]. Considering these findings, should miR159 be termed as negative or positive regulator in controlling the vegetative-to-reproductive transition? These conflicting reports might be explained if we assume that miR159 has multiple targets that are involved in different regulatory pathways, controlling flowering time, and the downstream targets of miR159 could be negative or positive regulators of flowering. miR159 was also involved in flower, embryo, seed and fruit development in plants. In *Larix kaempferi*, miR159 and its target *MYB33* showed opposite expression trends during somatic embryo maturation, suggesting that miR159 regulates the developmental transition of the embryo by repressing *MYB33* expression [36]. In *Brassica napus*, miR159 was strongly expressed at later stages of seed development, and its target, *MYB101*, exhibited a contrasting expression trend that was expressed at very low levels throughout seed maturation [37], which shows that the miR159-*MYB101* module is involved in the developmental transition of seeds. The miR159-*MYB101* module also controlled stress-induced premature transition to the reproductive phase in *Arabidopsis* [38]. In tomato, miR159-overexpressing plants exhibited precocious fruit initiation and obligatory parthenocarpy accompanied by *MYB1/2* silencing, which reveals that miR159-*MYB1/2* modules regulate the transition from flowering to fruit production, which is crucial to ensure successful sexual plant reproduction [39]. Moreover, *MYB1/2* silencing in miR159-overexpressing plants resulted in abnormal fruit development [39]. A recent report revealed that the miR159-*MYB* module was involved in the development of pollen in *B. campestris*, and the overexpression of miR159a caused pollen abortion, accompanied with the downregulation of two targets (*MYB101/120*) [40].

MiR159 has also been demonstrated to be involved in the transition of the juvenile-to-adult phase, independently of GA. In *Arabidopsis*, the *mir159ab* double mutant had a significantly faster rate of leaf initiation and much smaller leaves than wild-type plants [41]. *mir159ab* also produced abaxial trichomes later than wild-type plants, whereas miR159a-overexpressing plants produced abaxial trichomes significantly earlier than normal [41]. These findings suggest that miR159 could act as a positive regulator in the juvenile-to-adult transition of plants. However, the miR159 was identified as a post-transcriptional negative regulator in the root growth of *Arabidopsis* [42]. The *mir159ab* double-mutant generated a larger meristem in root than wild type and formed longer roots, accompanied by the increased expression of three targets, *MYB33*, *MYB65* and *MYB101* [42].

In one of our recent studies, miR159 targeted *MYB29* and the upregulation of miR159 expression corresponded well to the downregulation of *MYB29* expression during the aerial stem-to-rhizome transition of *G. pentaphyllum* (unpublished data). A previous report indicates that the overexpression of a *B. oleracea MYB29* enhanced the accumulation of aliphatic glucosinolates (GSLs) in *Arabidopsis* [43]. GSLs contain three classes of aromatic, aliphatic, and indolic GSLs that are regulated by different members of the *MYB* family, and *MYB29* is responsible for the biosynthesis of aliphatic GSLs [44]. GSLs’ profiles differ extensively within and across plant tissues [45]. In *B. rapa ssp. Pekinensis*, aliphatic GSLs were mainly accumulated in the stem, whereas aromatic GSLs were mainly accumulated in the roots, which correlated well with the much higher expression of *MYB29* in the stem than in the other organs [44]. The variation in the level and composition of GSLs can affect plant growth, fitness, and yield [46], implying that different GSLs have different regulatory mechanisms to adapt developmental and environmental changes. Considering that the stem could acquire the characteristics of the root during the aerial stem-to-rhizome transition of *G. pentaphyllum*, it is thus suggested that the upregulation of miR159 expression reduced the level of aliphatic GSLs by repressing *MYB29* expression, to adjust growth in response to aerial stem-to-rhizome transition in *G. pentaphyllum*.

## 3. miR166

To date, cumulative data suggest that miR166 is primarily involved in the developmental transitions within vegetative or reproductive growth, but not in the vegetative-to-reproductive transition in plants. MiR166 regulates various developmental processes, such as SAM maintenance, root, stem, leaf, flower, and seed development, as well as rhizome formation [7,47,48,49,50].

In *Arabidopsis*, the miR166 family has multiple members, and all the family members (except for miR166g) were highly expressed in the SAM and root–hypocotyl junctions, and moderately expressed in the cotyledon vasculatures of young seedlings. As the plant grows, the expression regions were rapidly spread to the leaf veins and root tissues, with the highest expression in the root tip. In contrast, miR166g was not detected in the SAM, but highly expressed in rosette leaf trichomes and moderately expressed in root tissues [48]. These findings suggest that miR166 family members have spatiotemporal specificity in regulating plant development. MiR166 was found to target several members of *Homeodomain Leucine Zipper Class III* (*HD-ZIP III*) gene family, including *PHABULOSA* (*PHB*), *PHAVOLUTA* (*PHV*), *REVOLUTA* (*REV*), *ARABIDOPSIS THALIANA HOMEOBOX 8* (*ATHB8*) and *ATHB15* in *Arabidopsis* [50]. The overexpression of miR166g significantly reduced the expression of *PHB*, *PHV* and *ATHB15*, resulting in enlarged shoot meristems, radialized leaves, vascular defects and reduced gynoecia [51], suggesting that miR166g affected the normal transitions within organ development. The elevated miR166a level also reduced *PHB*, *PHV* and *ATHB15* expression, leading to growth retardation at seedling stage and even the absence of SAM in *Arabidopsis* [48], indicating that miR166a and its targets inhibited the transition of juvenile-to-adult phase. The phenotypic differences between transgenic *Arabidopsis* overexpressing miR166a and miR166g were probably due to their spatiotemporal specificity, mentioned above. In *Medicago truncatula*, the overexpression of miR166a decreased the ability of the root to form lateral root by repressing *MtCNA1*, *MtCNA2*, and *MtHB8*, the three homologs of *ATHB15* and *ATHB8* [52], suggesting that miR166a could be a regulator of lateral organogenesis in plants. Moreover, the miR166a-dependent regulation of the three targets controlled vascular bundle patterning in the root [52]. In poplar, miR166 was found to be involved in the development of stem vascular tissue [53]. The populus *POPCORONA* (*PCN*) gene is a homolog of *ATHB15*. The transgenic poplar overexpressing a miR166-resistant *PCN* delayed the differentiation of secondary xylem and phloem fiber [53], implying that miR166 could promote the secondary growth of woody stems by inhibiting *PCN* expression, and thus regulate the transition from the primary growth phase to the secondary growth phase. In the secondary growth of woody plants, the production of vascular tissue is derived from the activity of vascular cambium [54]. MiR166 transcripts were found to change in vascular cambium from dormancy to active growth in *Cunninghamia lanceolate* [55], suggesting that miR166 could regulate the transition of vascular cambium activity and thus affect wood formation. MiR166-regulated developmental transition of leaves was subsequently found in monocot plants. In rice, the downregulation of miR166 and the up-regulation of its several targets (*HD-ZIP III* genes) caused by the overexpression of a *trans*-acting siRNA (siR2141) disturbed leaf polarity and vascular development, and thus retarded growth stage transition [56]. In wheat, the silencing of miR166 led to the upregulation of its target gene *HD-ZIP HOX33-like*, and generated a leaf-twist phenotype in seedlings [57]. In maize, miR166h was significantly upregulated in the elongation zone of young leaves, suggesting that miR166h possibly control the transition of cell division-to-cell expansion during leaf development [58]. MiR166-mediated developmental transition was further confirmed in the formation of reproductive organs in *Arabidopsis* and other dicot plants. In developing anthers of *Arabidopsis*, miR166 promoted the expression of *SPOROCYTELESS/NOZZLE* (*SPL/NZZ*), a regulator of sporocyte development, by inhibiting the expression of *PHB*, to allow floral cells into sporocytes [59]. MiR166 was also found to be involved in the developmental transition of embryo. In *Pinus taeda*, the expression of miR166 reached its highest level in the female gametophyte during zygotic embryo development, when expanded cotyledons enclosed the SAM, which is a critical transition point where somatic embryo maturation often stops, indicating that miR166 played an important role in sustaining a maturing embryo [4]. Also, in transgenic *L. leptolepis*, miR166a was significantly upregulated as the somatic embryo matured, suggesting that miR166 regulated the transition from early to late development phases of embryo [60]. Considering these observations, miR166 appears to be an activator of embryo maturation. However, the specific loss of miR166 and ectopic expression of two miR166 targets, *PHB* and *PHV*, activated related seed maturation genes to promote seed maturation in *Arabidopsis* [49], implying that miR166 seems to be an inhibitor of embryo maturation. These conflicting reports imply that, besides *HD-ZIP* genes, miR166 might target other genes to regulate the developmental transitions during embryo maturation. 

In our studies, two members of miR166 family, miR166b and miR166e, were found to be involved in the aerial stem-to-rhizome transition in *G. pentaphyllum* [7]. MiR166b was predicted to target an *enoyl-CoA hydratase 2* (*ECH2*) gene and they exhibited significantly opposite expression trends during the aerial stem-to-rhizome transition [7]. *ECH2* was revealed to participate in IBA-to-IAA conversion by promoting β-oxidation of IBA in *Arabidopsis* [61]. The long-standing acid growth theory postulates that IAA could enable cell expansion in shoots by triggering cell-wall acidification, and thus activating cell wall-loosening enzymes [62]. *ECH2* likely promoted post-mitotic cell expansion during the cotyledon development in *Arabidopsis* [63]. The target of miR166e was a putative scopoletin glucosyltransferase (*SGT*)-like gene, and the downregulation of miR166e well corresponded to the upregulation of its target [7]. *SGT* enzyme catalyzes the formation of scopolin [64], which inhibited IAA catabolism during tobacco seedling development [65]. Thus, it was hypothesized that the miR166b-*ECH2* and miR166e-*SGT-like* modules probably synergistically promote cell expansion by converting IBA to IAA and preventing the degradation of IAA during the aerial stem-to-rhizome transition of *G. pentaphyllum* [7]. 

## 4. miR156

MiR156 is highly expressed at seedling stage and downregulated with age, acting as a master negative regulator of juvenile-to-adult and vegetative-to-reproductive transitions in plants [1,10,66,67]. 

In plants, the function of miR156 to regulate developmental phase transitions was originally confirmed in *Arabidopsis*, which was achieved by targeting the expression of *SQUAMOSA PROMOTER BINDING PROTEIN LIKE* (*SPL*) genes [33,68]. *SPL* was first identified in *Antirrhinum majus*, encoding a plant-specific transcription factor with a conserved *SBP* domain, by which *SPL* can recognize and bind specifically to the promoter region of downstream target genes to regulate plant development [69,70,71]. The *SPL* family contains multiple members, such as 16, 19, 35, 28, 13 members in *Arabidopsis* [72], rice [73], switchgrass [74], poplar [75], and *Cleistogenes songorica* [76], respectively. Some of them were found to be involved in the transition of developmental phases mediated by miR156. In *Arabidopsis*, the overexpression of miR156b produced more leaves and delayed flowering, accompanied by the downregulation of multiple *SPL* genes (*SPL2/3/4/5/6/9/10/11/13/15*) [33]. Contrarily, the decrease in the level of miR156a/miR156 caused the upregulation of several *SPL* genes, thereby producing less leaves and promoting juvenile-to-adult transition as well as flowering [68,77]. A recent research revealed that the mutation of miR156c or double mutation of miR156a/c upregulated the expression of seven *SPL* genes (*SPL2/3/9/10/11/13/15*), thus accelerating the production of leaf trichomes and promoting the juvenile-to-adult transition in *Arabidopsis* [10]. In monocot rice, the miR156-*SPL14* module was found to be involved in the control of developmental phase transitions [78,79,80,81,82]. The overexpression of a miR156-resistant *SPL14* caused a decrease in tiller number, but an increase in plastochron and an acceleration of floral transition [80]. In transgenic rice overexpressing miR156f, the decreased expression of *SPL14* together with *SPL3* and *SPL12* produced more tillers and displayed dwarfism [81]. Another report indicated that the overexpression of two miR156 genes (miR156b/h) resulted in reduced panicle size, and, in particular, delayed flowering. Contrary to the expression trend of the miR156b/h, *SPL2/12/13* and *SPL16/18* showed decreased mRNA levels in the flag leaves and panicles of transgenic plants, respectively, while *SPL14* was downregulated in both flag leaves and panicles of transgenic rice [82]. The tissue-specific expression of miR156-targeted *SPL* genes was further confirmed in *Arabidopsis*. For example, *SPL9* was expressed in young leaf primordia, whereas *SPL15* was only expressed during early stages of inflorescence development in *Arabidopsis* [67,83]. The MiR156-*SPL* module regulates the developmental phase transitions that have successively been confirmed in other herbaceous plants, such as tomato, switchgrass, potato, alfalfa and *G. pentaphyllum* [6,7,84,85,86]. A similar mechanism for miR156-mediated developmental phase transitions was also found in perennial woody plants. In poplar, the overexpression of miR156 reduced the expression of *SPL3/9*, which drastically prolonged the juvenile phase [87]. Several members of miR156 family were downregulated in the vascular cambium from dormancy to activity in tree species *C. lanceolata* [55], suggesting that miR156 could regulate the dormancy-to-activity transition of vascular cambium in conifers. In the tree species *Fortunella hindsii*, the overexpression of miR156a significantly enhanced the somatic embryogenesis (SE) competence, accompanied by the downregulation of multiple *SPL* genes (*SPL3/5/6/13/14*), indicating that miR156-*SPL* module regulated the initial phases of SE induction [88]. A recent report indicated that miR156 was downregulated in the floral transition of the tree species *Castanea mollissima*, and most of the miR156-targeted *SPL* genes, particularly *SPL6/9/10/13/16*, were significantly upregulated [89]. These findings suggest that miR156 and its targets are evolutionarily conserved in regulating the developmental phase transitions in both annual herbaceous plants and perennial tree species.

It is worth noting that the overexpression of miR156 produced aerial tubers but reduced underground tuber yield under short day (SD) condition in potato, which was suggested to be associated with a threshold level of miR156 [6]. In wild-type potato, all axillary meristems have the capacity to form tubers, but this potential is suppressed except in underground stolons [90]. MiR156, as a phloem-mobile signal, is thought to be transported to the stolon through phloem to promote tuberization when the level of miR156 exceeds a certain threshold, which in turn reduces the miR156 accumulation in aboveground meristems [6]. Thus, it was suggested that the overexpression of miR156 resulted in levels above the threshold in all the aboveground axillary meristems, and thus produced aerial tubers under SD conditions; however, the reduced underground tuber yield was possibly attributable to the prolonged juvenile phase of these plants [6]. In our research, miR156a was first found to promote the aerial stem-to-rhizome transition by repressing the expression of *SPL6* and *SPL13A* in *G. pentaphyllum* [7].

Some regulatory factors have been found upstream of miR156 to regulate its expression, such as transcription factors, hormones, and light [2,7,41,89,91]. A photoperiod-related transcription factor, *PETER PAN SYNDROME* (*PPS*), regulated the onset of the adult phase and the time of flowering by promoting the expression of miR156 in rice [2]. Our recent studies have shown that the miR156a upstream sequence contains several light-regulated motifs, and the light-mediated regulation of miR156a is probably involved in the aerial stem-to-rhizome transition in *G. pentaphyllum* [7]. It was also found that *AGAMOUS-like AGL15/18* and *MYB33* promoted the transcription of miR156a and miR156c by directly binding to the promoters of the two miRNAs in *Arabidopsis* [41,91]. A recent report revealed that GA, as an important hormone, significantly increased the expression level of miR156 and decreased the expression of the targeted *SPL* genes in *C. mollissima* [89].

## 5. miR172

In contrast to miR156, miR172 expression is up-regulated as plants age and acts as a positive regulator to control the juvenile-to-adult and vegetative-to-reproductive transitions [1,6]. MiR172 regulates these developmental phase transitions, through targeting *APETALA2* (*AP2*) family members, including *AP2* itself, *TARGET OF EAT 1* (*TOE1*), *TOE2*, *TOE3*, *SCHLAFMUTZE* (*SMZ*) and *SCHNARCHZAPFEN* (*SNZ*) in *Arabidopsis* [1,92]. The overexpression of both miR172a and miR172b promoted the juvenile-to-adult transition and flowering by repressing the expression of their targets in *Arabidopsis* [83,92,93]. Conversely, the loss-of-function mutation in miR172 delayed the formation of adult characteristics such as leaf trichomes, thereby inhibiting the juvenile-to-adult transition in *Arabidopsis* [83]. A recent report revealed that miR172 and its two *AP2* targets were involved in the maintenance of stem cells in the shoot apex and regulated the juvenile-to-adult transition in woody poplar [94]. Accumulating evidence indicate that miR172 and its targets not only regulate the vegetative-to-reproductive transitions but also control developmental transitions in the formation of vegetative organs. In transgenic wheat overexpressing miR172d, the expression level of *AP2-5* homologs were decreased and resulted in earlier flowering and a longer stem internode than wild-type [95]. In tree species *Jatropha curcas*, the overexpression of miR172a not only resulted in early flowering but also affected the development of vegetative organs, including altered leaf morphology, enhanced xylem development and reduced phloem development [93]. The gain-of-function and loss-of-function of miR172a promoted or delayed flowering and also altered leaf morphology by down- or upregulating the expression of an *AP2-like* gene in *Sinningia speciosa* [96]. It was also found that miR172 can regulate the normal transition within reproductive organ development. The loss-of-function of miR172 by mimicry technology reduced fruit size and affect fruit valve growth in *Arabidopsis* [97]. In barley, the increased level of *AP2* homologs resulted in an abnormal, indeterminate spikelet in the *mir172* mutant [98]. Generally, the functions of miR172 and its targets are relatively conservative between herbaceous plants and perennial tree species.

MiR172 and its targets has also a role in promoting underground tuber formation. In transgenic potato, the upregulation of miR172 promoted tuberization under long day (LD) but had no significant effect on tuber induction under SD [99]. This finding could be explained by a model for the control of tuberization by several factors, including *Phytochrome B* (*PHYB*), miR172, *RELATED TO APETALA2 1* (*RAP1*) and *BELL5* (*BEL5*) [99]. In the model, miR172 could promote the expression of a tuberization promoter *BEL5*, by targeting a homolog of *AP2*, *RAP1*. Under LD conditions, PHYB might repress the movement of miR172 and *BEL5* mRNA from leaves to stolon, and thus inhibit the tuberization in wild-type potato. However, in the transgenic potato, the increased miR172 and *BEL5* levels override the inhibition caused by LD, thereby promoting tuberization. Under SD, this repression of PHYB would be released and allow miR172 and *BEL5* into stolon to induce tuberization [99]. These findings imply that miR172 and *BEL5* could promote tuberization only when their expression levels reach a certain threshold level under LD, but the tuberization could not be enhanced continually with the increase in miR172 and *BEL5* levels under SD.

The promoters of several miR172 family members were found to contain auxin response elements (AuxREs), in which miR172b, miR172c and miR172d contained 2, 2, 1 AuxREs, respectively, in *Arabidopsis* [97,100]. The *Auxin Response Factors* (*ARF6/8*) could recognize the AuxREs in the miR172c promoter to activate the expression of miR172c and thus promote fruit growth in *Arabidopsis* [97]. Another research indicated that *ARF* was significantly up-regulated during the stolon-to-rhizome transition in lotus [101]. Rhizome is derived from modified stems (stolon) and used for asexual propagation, the predominant propagation way in lotus. Taken together, this suggests that miR172 promotes the developmental transition of organ formation by upstream regulation of *ARF* in plants.

MiR172 seems to indirectly regulate the expression of miR156-targeted *SPL* genes to control the developmental transitions in plants. In the miR172d-overproducing *Arabidopsis*, the *SPL3/4/5* genes were up-regulated, which led to early flowering [102]. However, the expression of several *SPL* genes (*SPL3/13/15/23*) was also increased in barley *mir172* mutant and affected the normal development of spikelets [98]. These findings imply that there are positive and negative regulators that regulate *SPL* expression between miR172 and *SPL*, and miR172 could mediate these positive and negative regulators to regulate *SPL* expression, thus controlling vegetative-to-reproductive transition and organ development. MiR156, in turn, could regulate the expression of miR172 via *SPL* genes. An miR156-mediated *SPL9* bound directly to the miR172b promoter to activate the expression of miR172b in *Arabidopsis* and potato [6,83]. It was further confirmed in rice that miR156-mediated *SPL14* bound the promoters of miR172b and miR172d to positively regulate inflorescence meristem and spikelet transition and negatively control tillering [103]. 

## 6. miR396

MiR396 has been shown to play multiple roles during various developmental phases of plants by inhibiting different targets, including *GROWTH-REGULATING FACTOR* (*GRF*), *BASIC HELIX-LOOP-HELIX* (*bHLH*), and *SHORT VEGETATIVE PHASE* (*SVP*) genes [104,105,106].

*GRF1-3* primarily promotes the leaf and cotyledon growth by regulating cell expansion and also negatively regulates flowering and male fertility to some extent in *Arabidopsis* [104]. In transgenic plants overexpressing miR396a and miR396b, the expression of six *GRF* genes (*GRF1/2/3/7/8/9*) was reduced and the most prominent phenotype was the reduced leaf width [107]. Another report revealed that the overexpression of miR396 (a/b/c/e/h/i/k) not only produced smaller leaves, but also resulted in dwarf, shorter roots, smaller and fewer siliques and seeds, with the down-regulation of *GRF1/2/3/4/7/8/9* expression in *Arabidopsis*. However, only the overexpression of miR396a/i led to abnormal flower development [108]. These findings indicate that miR396 family members have redundant functions but exhibit functional divergence to a certain degree. A recent research revealed that miR396-*GRF* module was involved in the embryogenic developmental transition in *Arabidopsis*, and the transcripts of five *GRF* genes (*GRF1/4/7/8/9*) were significantly up-regulated in the *mir396a/b* mutants, but were down-regulated in the miR396b overexpressor line during SE induction via an auxin-related pathway [109]. MiR396 family members played similar roles in other dicot plants, such as tobacco and tomato. In tobacco, the overexpression of miR396a down-regulated three *GRF-like* genes and resulted in narrow leaves, the abnormal development of stamens and pistils, and declined fertility with little or no seed production [110]. In tobacco, the overexpression of a *P. trichocarpa* miR396c repressed the expression of several *GRF* genes (*GRF1/3/8*) and led to a lack of SAM, as well as altered floral organ specification [111]. In tomato, the significant downregulation of miR396a and miR396b corresponded to a general upregulation of three *GRF* genes (*GRF1/2/5*), and produced bigger flowers, in particular, sepals and fruits [112]. These findings suggest that miR396 family members primarily regulate developmental transitions during the growth of vegetative and reproductive organs in dicot plants. Similar findings were also observed in monocot plants such as rice and maize. The overexpression of miR396d in rice significantly reduced the expression of several *GRF* genes (*GRF1/2/3/4/5/6/7/8/10*), and gave rise to defects in floral organ development, including open husks, long sterile lemmas similar to the phenotype of the *grf6* mutant [113]. In maize, miR396 expression was gradually decreased with advancing grain filling, which was significantly negatively correlated with the increased expression of *GRF* genes (*GRF1/6*) [114]. Later, the miR396-*GRF* module was found to not only control the normal transition of reproductive organ development but also regulate the vegetative organ development in rice. The overexpression of rice miR396a significantly downregulated several *GRF* genes (*GRF1/2/6/8*), resulting in dwarf, smaller leaves, abnormal panicles and spikelets, especially a large proportion of rare conjoined-twin florets [115]. These findings demonstrate the functional conservation of the miR396-*GRF* module across dicot and monocot plants. Recently, our study revealed that the expression of miR396b was up-regulated, whereas its target *GRF5-like* gene was down-regulated during the aerial stem-to-rhizome transition of *G. pentaphyllum* (unpublished data), suggesting that miR396 could promote rhizome formation by inhibiting its target *GRF* expression in plants. MiR396 and miR166 were speculated to interact through their targets, *GRF* and *HD-ZIP* genes. The target of miR166, *HD-ZIP* genes, can specify the adaxial differentiation of organs [116,117]. Interestingly, *GRF* genes, as the target of miR396, were found to have higher expression levels in the adaxial side of the leaf in *Arabidopsis*. Moreover, the expression level of *GRF* genes changed in the leaves with polar defects [118]. These findings suggest that *GRF* genes are downstream of genes involved in the dorsoventral axis formation, such as *HD-ZIP* genes [118]. In maize, miR396 and miR166 were found to have similar expression patterns, in that their expression levels were gradually decreased whereas the levels of their target genes, *GRF* and *HD-ZIP* homologs, were gradually increased during grain filling [114]. Taken together, the hypothesis that miR396 and miR166 might interact through their target genes seems to make sense.

*SVP*, as one of the target genes of miR396, was found to negatively regulate flowering, like *GRF* in *Arabidopsis*. The overexpression of *SVP* reduced the expression of a floral promoter *FLOWERING LOCUS T* (*FT*) and delayed flowering, whereas the *svp* mutant showed early flowering [119]. It is worth noting that the decreased expression of both *SVP* and *GRF1* also resulted in a late-flowering phenotype, with curved sepals and petals and enlarged stigmas in the transgenic *Arabidopsis* overexpressing AtmiR396a and *Catharanthus roseus* miR396 [105]. Thus, it is suggested that miR396 regulates the normal development of floral organs by targeting *SVP* and *GRF*, while it probably controls flowering time by regulating other targets.

MiR396 also regulates developmental transitions in the formation of vegetative organs by targeting *bHLH* genes. In *Arabidopsis*, *bHLH74* repression by miR396 was found to be required for leaf margin and vein pattern formation, and the overexpression of a miR396-resistant *bHLH74* gene resulted in alterations in leaf development, especially in the vein pattern and shape [106]. Further research revealed that the miR396-*bHLH* module was involved in root development. In *M. truncatula*, the overexpression of miR396b downregulated the expression of two *bHLH79-like* genes, resulting in a shorter root [120]. On the contrary, the overexpression of an miR396-resistant *bHLH74* produced longer roots in *Arabidopsis* seedlings [121]. These findings suggest that miR396 could repress the root growth by targeting *bHLH* genes. In a recent study, an *mir396a/b* double-mutant produced the first leaf with abaxial trichomes earlier than the wild type and exhibited early flowering in *Arabidopsis*, whereas the miR396a/b overexpression line showed opposite phenotypes [122]. Moreover, the expression of *bHLH74* and several *SPL* genes were significantly upregulated in the *mir396a/b* mutant [122]. Given that *bhlh74* showed a moderate late flowering phenotype [122] and *SPL* is a flowering activator, as mentioned above, it is suggested that miR396 could inhibit juvenile-to-adult and vegetative-to-reproductive transitions by directly targeting *bHLH74* and indirectly repressing *SPL* expression. 

## 7. Conclusions

In recent years, molecular genetic analysis regarding miRNAs controlling plant developmental transitions has produced significant advances, and five miRNA families (miRNA159/166/156/172/396) have been demonstrated to be key regulators in the developmental transitions of plants (Figure 1, Figure 2 and Figure 3; Table S1). MiR156, miR172, and miR159 specifically target the members of *SPL*, *AP2*, *MYB* family, respectively. MiR172 promotes juvenile-to-adult and vegetative-to-reproductive transitions, whereas miR156 shows opposite regulatory roles. Recent research reveals that the two miRNAs, in particular miR172, also play a role in the normal transitions within the growth of vegetative or reproductive organs. Similar to miR172, miR159 can also promote juvenile-to-adult transition, but controlling flowering time is controversial. Unlike the above-mentioned three miRNAs, miR166 and miR396 have multiple targets, which mainly regulate the developmental transitions in organ formation. However, miR166-targets regulatory modules are functionally less conservative than miR396-targets modules. It is worth noting that the five miRNAs all regulate the developmental transition in the formation of storage organs (Figure 2). Among them, miRNA156, miR159, miR172, and miR396 are found to positively regulate aerial stem-to-rhizome or underground stolon-to-tuber transitions. while miR166 negatively controls these transitions. In general, the functions of the five miRNAs and their targets are relatively conserved, at least in seed plants, but functional divergences also emerge to varying degrees.

Current researches reveal that miRNAs usually negatively regulate their downstream targets to affect the developmental process of plants. Thus, it is possible to hypothesize that plants could break the existing developmental status through the negative regulation of miRNAs on their target genes, to achieve developmental phase transitions. However, it is still poorly understood how miRNAs themselves are spatiotemporally regulated. More researches about the complex interplay of these miRNAs with other regulatory factors, in particular, acting upstream of miRNAs, would not only help address this issue but would also probably clarify some existing controversial issues, such as miR159-mediated flowering time and miR166-mediated embryo maturation. In addition, much still remains to be discovered about miRNAs-regulated developmental transitions in the formation of storage organs and vascular tissues, which would be an important molecular basis for improving crop yield and tree biomass through genetic manipulation. It is worth noting that, although gene overexpression helps to understand the role of the gene-of-interest in many plants, especially in non-model plants, the transgenic plants overexpressing a gene can present pleiotropic phenotypes and sometimes may not well elucidate the function of the gene. Therefore, more mutant analysis and the application of gene editing technology, such as CRISPR-Cas9, might provide new insights into plant developmental phase transitions regulated by miRNAs and their targets in the future.

## Supplementary Materials

Supplementary materials can be found at https://www.mdpi.com/1422-0067/21/6/1971/s1.

## Abbreviations

miRNAs	MicroRNAs
SAM	Shoot apical meristem
GA	Gibberellin
GSLs	Glucosinolates
HD-ZIP III	Homeodomain leucine zipper class III
PHB	Phabulosa
PHV	Phavoluta
REV	Revoluta
ATHB8	*Arabidopsis thaliana* homeobox 8
ATHB15	*Arabidopsis thaliana* homeobox 15
PCN	Popcorona
SPL/NZZ	Sporocyteless/Nozzle
ECH2	Enoyl-CoA hydratase 2
SGT	Scopoletin glucosyltransferase
SPL	Squamosa promoter binding protein like
SD	Short day
PPS	Peter pan syndrome
AP2	Apetala2
TOE1	Target of eat 1
SMZ	Schlafmutze
SNZ	Schnarchzapfen
LD	Long day
PHYB	Phytochrome B
RAP1	Related to apetala2 1
BEL5	Bell5
AuxREs	auxin response elements
ARF	Auxin response factors
GRF	Growth-regulating factor
bHLH	Basic helix-loop-helix
SVP	Short vegetative phase
FT	Flowering locus T
