# Supplementary material for: The Control of Developmental Phase Transitions by microRNAs and Their Targets in Seed Plants
Source: Int J Mol Sci. 2020 Mar 13;21(6):1971. doi: 10.3390/ijms21061971 (PMC7139601; doi:10.3390/ijms21061971)
Supplement: Supplementary file 1 [file ijms-21-01971-s001.pdf]

Table S1. MiRNAs and their targets involved in the regulation of developmental phase transitions and storage organ formation.

MiRNAs	Targets	Functions	Species	References
miR159	*MYB65*	Flowering time/Root growth	*Arabidopsis*	34, 42
	MYB101	Stress-induced premature transition to reproductive phase/Root growth	*Arabidopsis*	38, 42
		Embryonic development	*Brassica napus*	37
		Pollen development	*Brassica campestris*	40
	MYB33	Flowering time/Anther defects/Juvenile-to-adult phase transition/Root growth	*Arabidopsis*	30, 33, 34, 41, 42
		Embryonic development	*Larix kaempferi*	36
	MYB120	Pollen development	*Brassica campestris*	40
	MYB	Flowering time	Gloxinia	31
			Rice	32
	MYB29	Aerial stem-to-rhizome transition	*Gynostemma pentaphyllum*	Unpublished data
	MYB33-like	Flowering time	Tobacco	35
	MYB1/2	Flowering to fruit production transition/Abnormal fruit development	Tomato	39
miR166	*PHB*	Organ development/ Growth retardation/Anther development/Seed maturation	*Arabidopsis*	48, 49, 51, 59
	PHV	Organ development/ Growth retardation/Seed maturation	*Arabidopsis*	48, 51, 49
	ATHB15	Organ development/Growth retardation	*Arabidopsis*	48, 51
	ECH2	Aerial stem-to-rhizome transition	*Gynostemma pentaphyllum*	Unpublished data
	GT-like	Aerial stem-to-rhizome transition	*Gynostemma pentaphyllum*	Unpublished data
	MtCNA1	Lateral root organogenesis/ Vascular bundle patterning	*Medicago truncatula*	52
	MtCNA2			
	MtHB8			
	PCN	Stem development	Poplar	53
	HD-ZIP III	Leaf polarity/Vascular development	Rice	56
	HD-ZIP HOX33-like	Leaf development	Wheat	57
		Vascular cambium from dormancy to active growth	*Cunninghamia lanceolate*	55
		Somatic embryo development	*Larix leptolepis*	60
		Leaf development	Maize	56
		Zygotic embryo development	*Pinus taeda*	4
miR156	*SPL4*	Leaf development/ Flowering time	*Arabidopsis*	33
	SPL11	Leaf development/ Flowering time/ Juvenile-to-adult transition	*Arabidopsis*	9, 33
	SPL15	Leaf development/ Flowering time/ Juvenile-to-adult transition	*Arabidopsis*	9, 33
	SPL	Juvenile-to-adult transition /Flowering time	*Arabidopsis*	68, 77
	SPL10	Leaf development/ Flowering time/ Juvenile-to-adult transition	*Arabidopsis*	9, 33
		Floral transition	*Castanea mollissima*	89
	SPL5	Leaf development/ Flowering time	Arabidopsis	33
		Somatic embryogenesis	*Fortunella hindsii*	88
	SPL3	Leaf development / Flowering time/ Juvenile-to-adult transition	*Arabidopsis*	10, 33
		Juvenile-to-adult transition	Poplar	87
	SPL2	Leaf development / Flowering time/ Juvenile-to-adult transition	*Arabidopsis*	10, 33
		Organ development/ Flowering time	Rice	82
	SPL9	Leaf development/ Flowering time/ Juvenile-to-adult transition	*Arabidopsis*	9, 33
		Floral transition	*Castanea mollissima*	89
		Juvenile-to-adult transition	Poplar	87
	SPL6	Leaf development/ Flowering time	*Arabidopsis*	33
		Floral transition	*Castanea mollissima*	89
		Somatic embryogenesis	*Fortunella hindsii*	88
		Aerial stem-to-rhizome transition	*Gynostemma pentaphyllum*	Unpublished data
	SPL13	Leaf development/ Flowering time/ Juvenile-to-adult transition	*Arabidopsis*	9, 33
		Somatic embryogenesis	*Fortunella hindsii*	88
		Organ development/ Flowering time	Rice	84
		Floral transition	*Castanea mollissima*	89
	SPL16	Floral transition	*Castanea mollissima*	84

		Organ development/ Flowering time	Rice	89
	SPL14	Somatic embryogenesis	*Fortunella hindsii*	88
		Organ development/ Flowering time	Rice	84
		Tiller development/ Dwarf		81
	SPL13A	Aerial stem-to-rhizome transition	*Gynostemma pentaphyllum*	Unpublished data
	SPL12	Organ development/ Flowering time	Rice	84
		Tiller development/ Dwarf		81
	SPL18	Organ development/ Flowering time	Rice	84
miR172	*SPL4*	Flowering time	*Arabidopsis*	102
	SPL5			102
	SPL3	Flowering time	*Arabidopsis*	102
		Organ development	Barley	98
	AP2 homologs	Organs development	Barley	98
	SPL13	Organ development	Barley	98
	SPL15			98
	SPL23			98
	AP2-like	Organs development/ Flowering time	*Jatropha curcas*	93
			Sinningia speciose	96
	AP2	SAM maintenance/ Juvenile-to-adult transition	Poplar	94
	RAP1	Underground tuber formation	Potato	99
	AP2-5 homologs	Vegetative organs development/Flowering time	Wheat	95
		Juvenile-to-adult transition /Flowering time/Fruit development	*Arabidopsis*	83, 92, 93, 97
miR396	*GRF9*	Organ development/ Embryogenic developmental transition	*Arabidopsis*	107, 108, 109
	GRF7	Organ development/ Embryogenic developmental transition	*Arabidopsis*	107, 108, 109
		Floral organ development	Rice	113
	GRF4	Organ development/ Embryogenic developmental transition	*Arabidopsis*	107, 108, 109
		Floral organ development	Rice	113
	GRF3	Organ development	*Arabidopsis*	107, 108
			tobacco	111
		Floral organ development	Rice	113
	GRF2	Organ development	*Arabidopsis*	107, 108
		Organ development	Rice	113, 115
		Flower development	tomato	112
	GRF8	Organ development/ Embryogenic developmental transition	*Arabidopsis*	107, 108, 109
		Organ development	Rice	113, 115
		Organ development	tobacco	111
	GRF1	Organ development/ Embryogenic developmental transition	*Arabidopsis*	107, 108, 109
		Flowering time/Floral organ development		106
		Grain filling	maize	114
		Organ development	rice	113, 115
		Organ development	tobacco	111
		Flower development	tomato	112
	GRF5-like	Aerial stem-to-rhizome transition	*Gynostemma pentaphyllum*	Unpublished data
	GRF6	Grain filling	maize	114
		Organ development	rice	113, 115
	GRF10	Floral organ development	rice	113
	GRF5	Floral organ development	rice	113
		Flower development	tomato	112
	GRF-like	Organ development	tobacco	110
	SVP	Flowering time/Floral organ development	*Arabidopsis*	106
	bHLH74	Leaf development	*Arabidopsis*	105
		Root development		121
		Juvenile-to-adult transition/Flowering time		122
	bHLH79-like	Root development	*Medicago truncatula*	120
